# Evaluation of Annexins Family as Potential Biomarker for Predicting Progression and Prognosis in Clear Renal Cell Carcinoma

**DOI:** 10.1155/2022/8748434

**Published:** 2022-06-08

**Authors:** Jiyu Zhao, Luchen Chang, Jianping Tu, Bei Sun, Xi Wei

**Affiliations:** ^1^Department of Urology, ChuiYangLiu Hospital Affiliated to Tsinghua University, 100021 Beijing, China; ^2^Department of Diagnostic and Therapeutic Ultrasonography, Tianjin Medical University Cancer Institute and Hospital, National Clinical Research Center for Cancer, Key Laboratory of Cancer Prevention and Therapy, Tianjin's Clinical Research Center for Cancer, 300060 Tianjin, China; ^3^Department of Urology, The Third Hospital of Xiamen, 361199 Xiamen, Fujian, China; ^4^Department of Outpatient Office, Tianjin Medical University Cancer Institute and Hospital, National Clinical Research Center for Cancer, Key Laboratory of Cancer Prevention and Therapy, Tianjin's Clinical Research Center for Cancer, 300060 Tianjin, China

## Abstract

**Background:**

Annexins family (ANXAs), as a Ca^2+^-dependent phospholipid-binding protein superfamily, participates in a wide variety of biological activities and has been reported to be dysregulated in numerous types of human cancers. Evidence from cell lines and human tissues indicates that ANAXs are involved in kidney clear renal cell carcinoma (KIRC) tumorigenesis. However, their prognostic value and expression pattern associated with KIRC remain to be elucidated.

**Methods:**

We visited public databases, including ONCOMINE, Gene Expression Profiling Interactive Analysis (GEPIA), Kaplan–Meier plotter, cBioPortal, and GeneMANIA, to conduct comprehensive bioinformatics analysis and tried to detect basic relationships between each Annexins family member and KIRC.

**Results:**

We found that the expression level of ANXA1/2/4/5/6/7/8/13 in clear renal cell carcinoma tissue was higher than that in the kidney tissue, while the expression level of ANXA3/9/11 in the former was lower than that in the latter. The expression level of ANXA7/8/13 is related to the stage of the tumour. Survival analysis using the Kaplan–Meier plotter database showed that a high transcription level of ANXA2/5/8/10 is related to a low overall survival rate (OS) in predicting KIRC patients. In contrast, high ANXA3/4/7/9/11/13 levels are associated with a high OS in these patients.

**Conclusions:**

Our study implies that ANXA4/8/13 are potential targets of precision therapy for patients with KIRC and that ANXA2/5/8/10 are new biomarkers for the prognosis of KIRC.

## 1. Introduction

Renal cell cancer (RCC) is one of the most common malignancies, and approximately 73,820 patients were diagnosed in 2019 [1]. The majority of RCC patients have kidney clear renal cell carcinoma (KIRC) histology (75–80%), and the other common histological types include papillary, chromophobe, hereditary leiomyomatosis-associated RCC, and collecting duct carcinoma. These are called “nonclear cell cancers” [2]. Despite advancements in diagnostic methods and operative techniques that have allowed for more effective treatment of KIRC, the 5-year overall survival rate of metastatic KIRC remains less than 20% [1]. Therefore, it is necessary to investigate molecular markers to refine prognostic prediction and identify potential treatment targets.

Annexins are a Ca^2+^ dependent, phospholipid-binding protein superfamily, with members expressed in the zoology and botany cell. Annexins, including 12 family members, play a major role in the regulation of a broad range of physiological processes linked to cellular membranes [3]. Previous studies have revealed that the aberrant expression of ANXAs is frequently observed in various types of cancer, including cervical cancer [4], bladder cancer [5, 6], breast cancer [7–9], gastric cancer [10], lung cancer [11, 12], oral squamous cell carcinoma [13], hepatocellular carcinoma [14, 15], and cholangiocarcinoma [16]. ANXAs may function as either oncogenes or suppressors depending on tumour biology.

ANXA1/2/3/4/5/7 have been reported to be dysregulated in KIRC [17–20]. However, the majority of these studies only focused on the changes in expression levels and a few prognostic data available. The expression patterns, potential biological functions, and prognostic value of Annexins in KIRC have yet to be fully elucidated.

To the best of our knowledge, no bioinformatics analysis of Annexins profiles has been applied to investigate KIRC. The use of large-scale DNA/RNA sequencing has undergone revolutionary development and has become an integral part of biomedical research. In the present study, we performed a collective analysis of thousands of gene expression or variation in copy-number analyses published online to investigate the expression of Annexin family members in the database to determine its clinical value in KIRC.

## 2. Materials and Methods

### 2.1. Ethics Statement

This study was approved by the Academic Committee of ChuiYangLiu Hospital affiliated with Tsinghua University and performed in accordance with the ethical principles expressed in the Declaration of Helsinki. All data were collected from published literature.

### 2.2. ONCOMINE Analysis

The transcription levels of ANXAs in different cancers were analysed using the online tumour microarray database ONCOMINE (https://www.oncomine.org/). Comparison of ANXAs mRNA expression levels between tumour and normal samples was performed using Student's *t*-test. The *P* value and fold change were used as cut-off points at 0.0001 and 2, respectively.

### 2.3. Gene Expression Profiling Interactive Analysis (GEPIA) Dataset

We used the online database Gene Expression Profiling Interactive Analysis (GEPIA, https://gepia.cancer-pku.cn/) to analyse ANXAs mRNA sequencing expression data [21]. GEPIA could provide mRNA differential expression analysis according to cancer stage and patient survival data using one-way ANOVA. The analysis setting is |log_2_(FC)| ≥ 1 and *P* ≤ 0.01.

### 2.4. The Kaplan–Meier Plotter

We examined the prognostic value (OS and RFS) of ANXA mRNA expression in KIRC using the Kaplan–Meier plotter database [22] (https://www.kmplot.com/). We only selected the JetSet best probe set and automatically chose the best cut-off to perform the analysis.

### 2.5. cBioPortal

We examined the mutations and putative copy number alterations (CNA) of ANXAs in KIRC using cBioPortal (https://www.cbioportal.org/), an online database on 30 different cancers collected from The Cancer Genome Atlas [23]. A total of 317 KIRC cases with pathology reports were selected in our study. The online tools and data sources are provided by cBioPortal.

### 2.6. GeneMANIA

To explore interactive functional associations among ANXAs, we used GeneMANIA (https://www.genem/ania.org) to create an interactive functional-association network. The online tools and data sources are provided by GeneMANIA.

## 3. Results

### 3.1. Transcriptional Levels of ANXAs in Patients with KIRC

**Figure 1:**
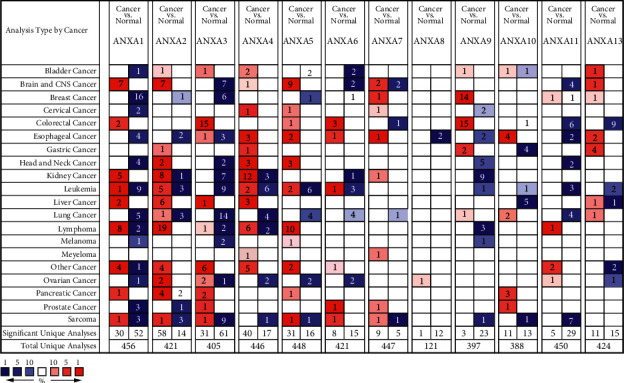
The transcription levels of ANXAs in different types of cancers (ONCOMINE). The threshold was set to following parameters: fold change = 2 and *P*-value = 0.0001. The cell number indicates the number of datasets that meets the thresholds. The color red or blue directly indicates up- or downregulation, respectively.

Twelve ANXA factors have been found in mammalian cells. We used the ONCOMINE database to compare the transcription levels of ANXA in cancer with those in normal samples (Figure 1). The mRNA expression levels of ANXA4 were significantly upregulated in patients with KIRC in twelve datasets. In Higgins's dataset [24], ANXA4 was overexpressed compared with that in normal samples in KIRC, with a fold change of 3.662. In Gumz's dataset [25], ANXA4 was also overexpressed in KIRC with a fold change of 13.931. The transcription levels of ANXA4 in KIRC are higher than those in normal tissues in Yusenko [26] and Jones's datasets [27], and their fold changes are 4.763 and 2.598, respectively (Table 1).

Higgins [24] showed another mRNA expression factor with increased expression; that is, ANXA2 has a fold change of 2.358 in patients with kidney renal clear cell carcinoma compared with that in patients with normal kidney tissues. ANXA2 overexpression is also found in kidney renal clear cell carcinoma, with a fold change of 2.444 in Gumz's dataset [25] and 2.383 in Jones's dataset [27] (Table 1).

The mRNA expression levels of ANXA1 and ANXA7 were upregulated in patients with KIRC. The transcription level of ANXA1 in KIRC is higher than that in kidney tissues, and their fold changes are 4.853, 5.348, and 3.26 in Yusenko [26], Gumz [25], and Beroukhim's datasets [28], respectively. In Jones's datasets [27], the mRNA expression of ANXA7 in KIRC increased by 2.565-fold.

### 3.2. Relationship between the mRNA Levels of ANXAs and the Clinicopathological Characteristics of Patients with KIRC

**Figure 2:**
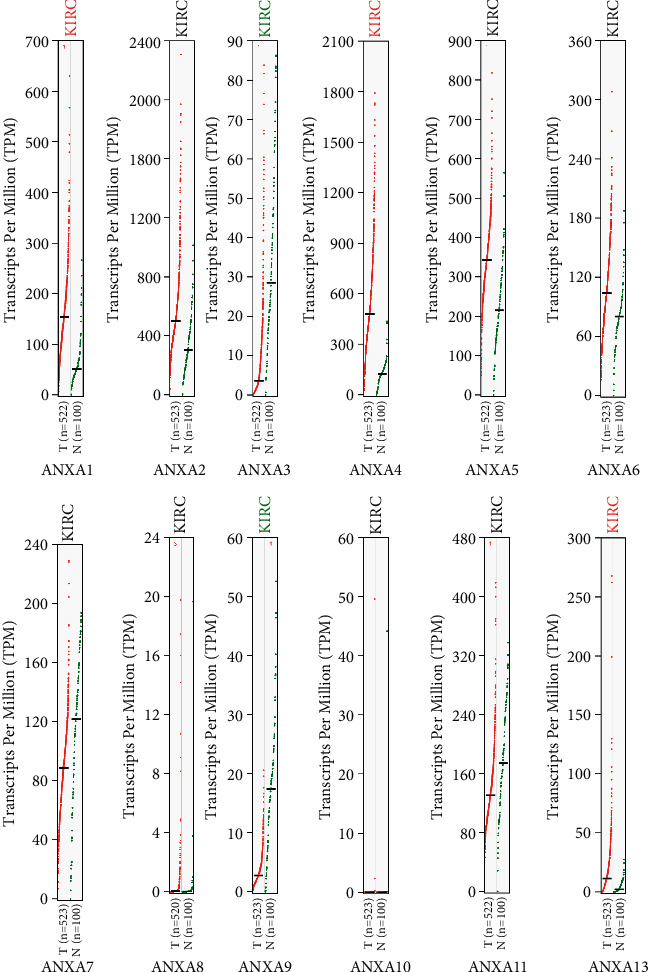
The mRNA expression levels of ANXAs in clear renal cell carcinoma and normal kidney tissues (GEPIA). The plots show mRNA expression of Annexins in kidney tumour (red plot) and the corresponding expression in normal tissues (green plot).

**Figure 3:**
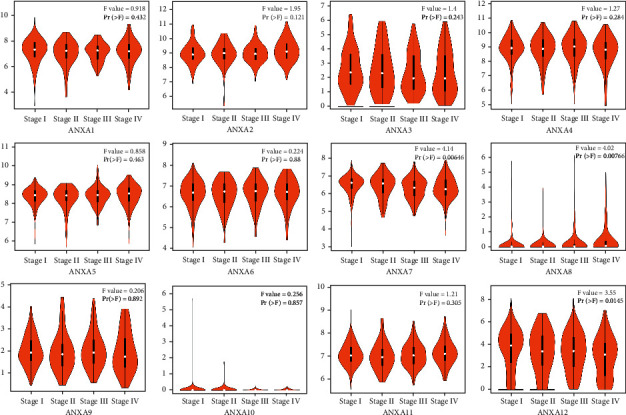
Correlation analysis of the ANXAs expression and clinical stages in clear renal cell carcinoma (GEPIA). Spearman's correlation analysis between gene and clinical stages is based on the entire clinical stages.

Using the GEPIA (Gene Expression Profiling Interactive Analysis) dataset (https://gepia.cancer-pku.cn/), we compared the mRNA expression of ANXAs factors between KIRC and kidney tissues. The results indicated that the expression levels of ANXA1, ANXA2, ANXA4, ANXA5, ANXA6, ANXA8, and ANXA13 were higher in KIRC tissues than in kidney tissues, whereas the expression levels of ANXA3, ANXA7, ANXA9, and ANXA11 were lower in the former than in the latter (Figure 2). We also analysed the expression of ANXAs with tumour stage for KIRC. The ANXA7, ANXA8, and ANXA13 groups significantly varied, whereas the ANXA1, ANXA2, ANXA3, ANXA4, ANXA5, ANXA6, ANXA9, ANXA10, and ANXA11 groups did not significantly differ (Figure 3).

### 3.3. Association of the Increased and Decreased mRNA Expression of ANXAs with the Improved Prognosis of Patients with KIRC

**Figure 4:**
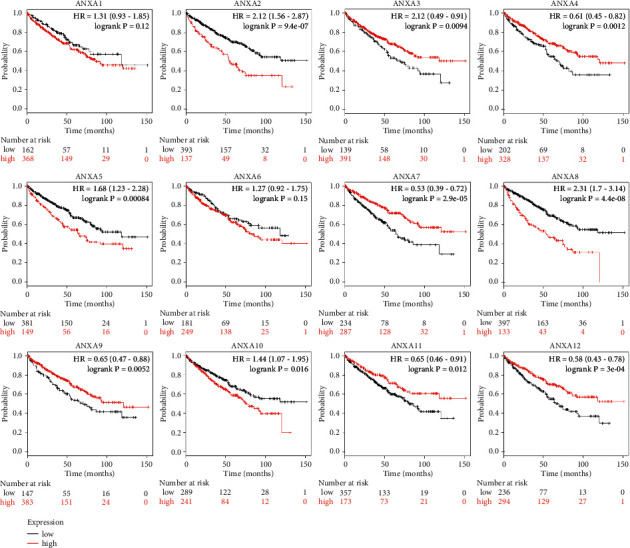
Prognostic values of ANXAs in clear renal cell carcinoma (overall survival in Kaplan–Meier plotter). The *P*-values were calculated using the log-rank test.

**Figure 5:**
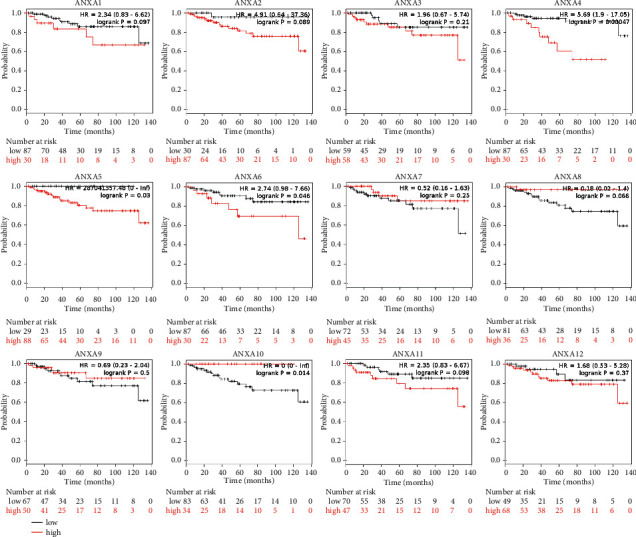
Prognostic values of ANXAs in clear renal cell carcinoma (relapse-free survival in Kaplan–Meier plotter). The *P*-values were calculated using the log-rank test.

We further explored the critical efficiency of ANXAs in the survival of KIRC patients. We applied the Kaplan–Meier plotter tool using a publicly available dataset (https://kmplot.com/analysis/index.php?p=service&cancer=pancancer_rnaseq) to analyse 530 KIRC patients for ANXAs correlation between mRNA levels and survival. Analysis by Kaplan–Meier curve and log-rank test revealed that increased levels of ANXA2/5/8/10 mRNA and decreased levels of ANXA3/4/7/9/11/13 mRNA in KIRC patients were significantly associated with decreased overall survival (OS) (*P* < 0.05) (Figure 4). KIRC patients with higher levels of ANXA1/4/5/6 mRNA or lower levels of ANXA10 mRNA were predicted to have lower relapse-free survival (RFS) (*P* < 0.05) (Figure 5).

### 3.4. Amplification, Deletion, Mutation, and Fusion of ANXAs in KIRC

**Figure 6:**
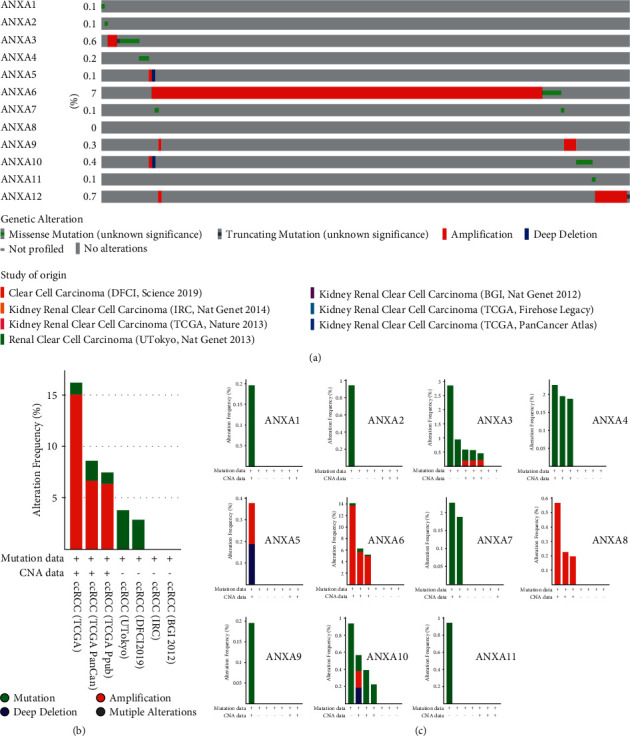
Analyses of genetic variations in ANXAs in clear renal cell carcinoma (cBioPortal). (a) OncoPrint visual summary of variations on a query of Annexin family members and overview of the analyses of genetic variations in ANXAs. (b) Analyses of genetic variations in Annexin family members reported in different studies. (c) Analyses of genetic variations in Annexin family members respectively in different studies.

Genetic variations of Annexins in 317 cases retrieved from four studies, including 35 cases from Dana-Farber Cancer Institute [29] (DFCI), 98 cases from Beijing Genomics Institute [30] (BGI), 78 cases from Cancer Research UK London Research Institute [31] (IRC), and 106 cases from the University of Tokyo [32] (Utokyo), were analysed using the cBioPortal database (Figure 6). We found varying degrees of genetic variation among the 12 ANXA family members, of which ANXA6 has the highest incidence of genetic variation. Most genetic variations in Annexins were amplifications, although ANXA3, ANXA4, and ANXA10 had higher probabilities of mutation events. Deep deletions were found in ANXA5 and ANXA10. In addition, no gene fusion events were found in the four datasets.

### 3.5. Correlations between ANXAs and Construction of a Gene-Gene Interaction Network

**Figure 7:**
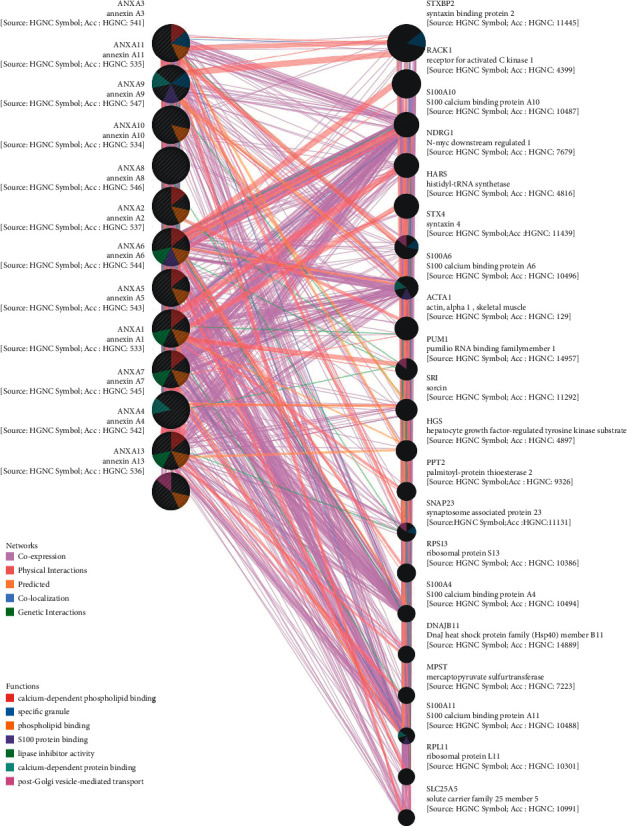
Gene-gene interaction network among Annexin family members (GeneMANIA). Each node represents a gene. The node size represents the strength of interactions. The connection lines represent the types of gene-gene interactions, and the line color represents the types of interactions. The node color represents the possible functions of respective genes.

The constructed functional network based on the gene function predictions of the 12 ANXAs through the GeneMANIA database is shown in Figure 7. The 12 central nodes representing ANXAs were surrounded by genes that were strongly correlated with physical interactions, coexpression, predictions, colocalization, and genetic interactions.

We established a gene-gene interaction network of 12 Annexin genes and analysed their functions through the GeneMANIA database (Figure 7). The top 5 genes representing the most relevant ones with the ANXAs included STXBP2 (syntaxin binding protein 2), RACK1 (receptor for activated C kinase 1), S100A10 (S100 calcium binding protein A10), NDRG1 (N-myc downstream regulated 1), and HARS (histidyl-tRNA synthetase). STXBP2 was correlated with ANXA3 in terms of colocalization and physically interacted with ANXA3 and ANXA11. RACK1 physically interacted with ANXA2. S100A10 was correlated with ANXA2 in terms of physical interactions, colocalized with ANXA5, and coexpressed with ANXA11, ANXA2, ANXA6, ANXA7, ANXA8, ANXA9, ANXA10, and ANXA11. NDRG1 physically interacted with ANXA5 and was coexpressed with ANXA3, ANXA4, and ANXA6. In addition, HARS was correlated with ANXA5 in terms of physical interactions and correlated with ANXA6 in terms of coexpression.

We also found that ANXAs had the greatest correlation with calcium-dependent phospholipid binding. Additionally, they were also correlated with specific granules, phospholipid binding, S100 protein binding, lipase inhibitor activity, secretory granules, regulation of vesicle-mediated transport, postGolgi vesicle-mediated transport, calcium-dependent protein binding, and enzyme-inhibitor activity.

## 4. Discussion

ANAXs, as a Ca^2+^-dependent, phospholipid-binding protein superfamily, have been reported in numerous types of human cancer and have participated in a wide variety of biological activities, such as tumorigenesis, progression, and resistance to chemotherapeutic agents [3, 33]. However, a further comprehensive bioinformatics analysis of ANAXs in KIRC has yet to be performed. In the present study, we analysed the relationship between the expression of different ANAX factors and prognoses (OS and RFS) of KIRC for the first time. We hope that our findings may help create a foundation to better understand and improve current therapies and prognostic accuracy for patients with KIRC.

ANXA1 is known as an anti-inflammatory protein but is recognized to have a broader role in tumour cell biology beyond inflammation alone. ANXA1, located on human chromosome 9q21.13, is the first characterized member of the superfamily [12]. Transposition of ANXA1, which was found in oesophageal cancer, could affect the activities of arachidonic acid metabolism [34]. However, it is more likely a double-edged sword due to its numerous and sometimes opposite functions. Yamanoi et al. [18] found that knockdown of ANXA1 inhibits the proliferation, migration, invasion, and adhesion of kidney carcinoma cells. We first used the Oncomine database to reveal that ANXA1 mRNA expression levels were significantly higher in KIRC tissues than in normal tissues. However, its expression did not correlate with specific clinical features in KIRC. Using the Kaplan–Meier plotter, we estimated the prognostic value of ANXA1 in patients with KIRC. Patients with KIRC with high mRNA levels of ANXA1 were predicted to have a lower RFS.

The ANXA2 monomer exists in the cell cytoplasm, and the heterotetramer complexed with S100A10, which exists on cell membranes. Sadashiv et al. [35] reported that moderate immune expression of ANXA2 was found in the proximal convoluted tubules, which were considered the origin of KIRC. Yang et al. [20] demonstrated that the migration and invasion abilities of tumour cells were suppressed by silencing ANXA2 expression, whereas tumour cell proliferation was not affected. In our study, the expression of ANXA2 in KIRC tissues was higher than that in normal tissues. We also demonstrated that high ANXA2 expression was significantly correlated with poor OS in patients with KIRC. However, its expression was not correlated with tumour stage.

Bianchi et al. [36] cultured primary cells from human renal cell carcinoma and observed two spliced isoforms of ANXA3. However, one spliced isoform of ANXA3 was downregulated in KIRC, and the other was upregulated. The total ANXA3 protein was downregulated in KIRC cultures based on microarray analysis. In our report, we demonstrated that the expression of ANXA3 was not significantly different between tumour and normal tissues, which seemed inconsistent with Bianchi et al. However, we found that high ANXA3 expression was significantly correlated with favourable OS in patients with KIRC.

A data-independent acquisition-mass spectrometry proteomic approach demonstrated that ANXA4 expression levels are higher in KIRC than those in normal tissues [37]. In our research, we proved for the first time that ANXA4 mRNA expression was higher in KIRC tissues than in normal tissues, but this expression did not correlate with tumour progression stage. Additionally, low ANXA4 expression was remarkably associated with poor OS in KIRC patients. This study suggests that ANXA4 acts as a tumour suppressor gene.

ANXA5 was upregulated in KIRC, and high expression was associated with a higher clinical stage and histological grade [19]. We found a similar result in our study; that is, the expression of ANXA5 in KIRC tissues was higher than that in normal tissues. Higher ANXA5 expression was significantly related to poor OS in patients with KIRC.

ANXA6 is highly expressed in a variety of tumours, such as acute myeloid leukaemia [38], bladder cancer [39], and breast carcinoma [40]. However, its expression and prognostic role in KIRC have not been reported. In the present study, we confirmed that ANXA6 expression was higher in KIRC tissues than that in normal tissues, but this expression was not related to the stage of the tumour. Higher ANXA6 expression was significantly correlated with poor RFS in patients with KIRC. Moreover, ANXA6 displayed the highest incidence rate of genetic variations among the super family members, which might be related to kidney tumour progression.

ANXA7 GTPase is considered a tumour suppressor frequently inactivated by genomic alterations at 10q21 in a variety of human malignancies, including KIRC [41]. In this report, we demonstrated that the expression of ANXA7 in KIRC tissues was higher than that in normal tissues in Jones's dataset [27], but we also obtained the opposite result from the GEPIA dataset. It was discovered that high-stage KIRC exhibited significantly reduced ANAX7 expression. Moreover, decreased ANXA7 mRNA levels were significantly associated with lower OS, which conformed to its role as a tumour suppressor.

ANXA8 is highly expressed in patients with several malignancies, such as ovarian cancer [42] and bladder cancer [43]. Harumi et al. [44] found that cotransfection with an expression vector for ANXA8 and a reporter gene vector containing the HIF-1*α* promoter enhanced the activity of the HIF-1*α* promoter. It might play a role in calcium fluctuation-mediated HIF-1*α* transcriptional activation in pancreatic cancer. However, ANXA8 expression and its prognostic role in KIRC have not been reported. In this report, we found that the expression of ANXA8 in KIRC tissues was higher than that in normal tissues, and high-stage KIRC exhibited significantly increased ANAX8 expression. Increased ANXA8 mRNA was significantly associated with poor OS.

ANXA9 and ANXA11 expression and prognostic roles in KIRC have not been reported. Yu et al. [45] demonstrated that ANXA9 promoted the invasion and metastasis of colorectal cancer and predicted poor prognosis. ANXA9 showed high expression in head and neck squamous cell carcinomas and was associated with the tumour differentiation grade. Hua et al. [46] found that ANXA11 participated in gastric cancer proliferation, migration, and invasion via the AKT/GSK-3*β* pathway. However, in our report, we demonstrated that the expression of ANXA9 and ANXA11 in KIRC tissues was lower than that in normal tissues, and the expression did not correlate with tumour stage. Decreased ANXA9 and ANXA11 mRNA levels were significantly associated with poor OS.

It has been proven that abnormal expression of ANXA10 plays a key role in the generation, progression, and prognosis of tumours [47–49], although its functional role remains to be clarified and has not been reported in KIRC. The decreased expression of ANXA10 probably participates in malignant progression and poor prognosis [50]. Overexpression of ANXA10 could promote apoptosis of hepatocellular carcinoma cells [51]. However, the results of the present study confirmed that high ANXA10 expression was significantly associated with poor OS. The expression of ANXA10 was not associated with clinical stage.

ANXA13 is the latest ANXA member to be identified. There are limited studies available that focus on ANXA13 in cancer. ANXA13 increases cell growth and invasion. It also portends lymph node metastasis and poor prognosis in human lung adenocarcinoma patients [52]. ANXA13 was identified as a regulator of chemotherapy resistance because ectopic overexpression of ANXA13 could increase the sensitivity of malignant breast cancer cells to rapamycin [53]. In the present study, the expression level of ANXA13 was higher in KIRC tissues than in kidney tissues, and high-stage KIRC exhibited significantly decreased ANXA13 expression. Moreover, the decreased ANXA13 mRNA level was significantly associated with poor OS.

Cancer cells carry different mutations, which leads to a wide variety of clinical manifestations. Many genes show variations in copy-number alterations and may be associated with recurrence and death [54]. In the present study, we found gene alterations in 9.32% of the Annexin family in the database, including an amplification rate of 7.77% and a mutation rate of 1.44%. The incidence rate of genetic variations in the ANXA6 gene was up to 7.0%, whereas ANXA3, ANXA4, and ANXA10 had higher probabilities of mutation events. However, our findings showed that KIRC has a relatively low alteration rate in ANXAs compared with other cancers [42, 55–57].

We adopted the GeneMANIA database to construct a gene-gene interaction network that clarified the mechanisms of function of ANXAs in kidney cancer. The results showed that 20 genes, including STXBP2, RACK1, S100A10, NDRG1, and HARS, were enriched in this network based on their functions associated with physical interactions, coexpression, colocalization, pathways, and genetic interactions. It has been demonstrated that RACK1 could regulate ANXA2 phosphorylation to make it involved in the invasion and metastasis of drug-resistant carcinoma cells. [58] NDRG1, like ANXAs involved in plasma membrane repair, has been recognized as a suppressor of carcinoma by decreasing EMT-associated protein expression [59]. Aberrant expression of these interacting genes is related to the tumorigenesis and progression of tumours, but these interactions with ANXAs in kidney cancer are intriguing and still need substantial experimental confirmation. It has been demonstrated that S100A10, located in the plasma membrane, can unite with ANXA2 to form a heterotetramer composed of two subunits, S100A10 and ANXA2. The heterotetramer could activate the plasminogen activation pathway, playing a key role in cellular repair and further promoting degradation of the extracellular matrix to increase the invasion capability of carcinoma cells [60].

## 5. Conclusion

In the present study, the expression of ANXAs was systemically analysed to evaluate their clinical and prognostic value in KIRC, which provided an important molecular biological basis for understanding the complex development of KIRC. The results indicated that ANXA1/2/4/7/13 may be potential therapeutic targets for KIRC treatment, whereas ANXA2/5/8/10 may be potential prognostic biomarkers of KIRC. The results of the present study introduced ANXA4/8/13 as good candidates for future experimental works.

## Figures and Tables

**Table 1:** The ANXA expression between different types of renal clear cell carcinoma and kidney tissues.

GENE	Cancer tissue vs normal tissue	Fold change	*t*-test	*P*-value	Dataset
ANXA1	Renal clear cell carcinoma vs kidney	4.853	11.741	1.18*E − *12	Yusenko et al.
	Renal clear cell carcinoma vs kidney	5.348	11.22	7.71*E − *10	Gumz et al.
	Renal clear cell carcinoma vs kidney	3.26	6.477	4.53*E − *06	Beroukhim et al.
ANXA2	Renal clear cell carcinoma vs kidney	2.358	10.276	3.49*E − *10	Higgins et al.
	Renal clear cell carcinoma vs kidney	2.444	9.038	2.17*E − *08	Gumz et al.
	Renal clear cell carcinoma vs kidney	2.383	10.586	9.99*E − *14	Jones et al.
ANXA3	NA	NA	NA	NA	NA
ANXA4	Renal clear cell carcinoma vs kidney	3.662	12.691	2.99*E − *12	Higgins et al.
	Renal clear cell carcinoma vs kidney	3.577	13.931	2.23*E − *11	Gumz et al.
	Renal clear cell carcinoma vs kidney	4.763	9.538	5.94*E − *08	Yusenko et al.
	Renal clear cell carcinoma vs kidney	2.598	8.071	1.12*E − *09	Jones et al.
ANXA5	NA	NA	NA	NA	NA
ANXA6	NA	NA	NA	NA	NA
ANXA7	Renal clear cell carcinoma vs kidney	2.565	7.775	5.98*E − *07	Jones et al.
ANXA8	NA	NA	NA	NA	NA
ANXA9	NA	NA	NA	NA	NA
ANXA10	NA	NA	NA	NA	NA
ANXA11	NA	NA	NA	NA	NA
ANXA13	NA	NA	NA	NA	NA

## Data Availability

The datasets generated during and/or analysed during the current study are available in the ONCOMINE database (https://www.oncomine.org/), Gene Expression Profiling Interactive Analysis (GEPIA, https://gepia.cancer-pku.cn/), the Kaplan–Meier plotter database, and cBioPortal (https://www.cbioportal.org/).

## Abbreviations

ANXAs: Annexins family
KIRC: Kidney clear renal cell carcinoma
GEPIA: Gene expression profiling interactive analysis
OS: Overall survival
RCC: Renal cell cancer
CNA: Copy-number alterations
RFS: Relapse-free survival
STXBP2: Syntaxin binding protein 2
RACK1: Receptor for activated C kinase 1
S100A10: S100 calcium binding protein A10
NDRG1: N-myc downstream regulated 1
HARS: Histidyl-tRNA synthetase.

## Additional Points

A preprint has been published in research square https://www.researchsquare.com/article/rs-837226/v1, “Evaluation of Annexins Family as Potential Biomarker for Predicting Progression and Prognosis in Clear Renal Cell Carcinoma [61],” but it is not published or under consideration by any journal.
